# Mucoepidermoid carcinoma of the retromolar trigone in an elderly female: A case report

**DOI:** 10.1016/j.amsu.2021.102487

**Published:** 2021-06-08

**Authors:** Rachid Aloua, Ulrich Opoko, Ouassime Kerdoud, Zahra Hmoura, Faiçal Slimani

**Affiliations:** a Faculty of Medicine and Pharmacy, Hassan II University of Casablanca, B.P 5696, Casablanca, Morocco; b Oral and Maxillofacial Surgery Department, CHU Ibn Rochd, B.P 2698, Casablanca, Morocco

**Keywords:** Malignant tumor, Mucoepidermoid carcinoma, Salivary gland tumors, Retromolar trigone

## Abstract

Tumors of the retromolar trigone area represent 12% of neoplasms of the oral cavity, and of which squamous cell carcinoma represents the main histological type. Mucoepidermoid carcinoma (MEC) of the accessory salivary glands is a rare entity, representing less than 5% of head and neck cancers, and 10% of all salivary gland tumors; its biological characteristics are very diverse, correlated to the histological grade of the tumor. This tumor has an excellent outcome after surgical management, with a prognosis related to the histological grade of the tumor. The authors reported a case of low-grade mucoepidermoid carcinoma, arising in the left retromolar trigon, in an 83-year-old woman who was treated by surgical removal of the tumor with a 1.5 cm border.

## Highlights

• Mucoepidermoid carcinoma (MEC) of the accessory salivary glands is a rare entity.
• Its biological characteristics are very diverse, correlated to the histological grade of the tumor.
• This tumor has an excellent outcome after surgical management; with a prognosis related to the histological grade of the tumor.
• We reported a case of low-grade mucoepidermoid carcinoma, arising in the left retromolar trigon, in an 83-year-old woman, treated by surgical resection.

## 1 Introduction

Mucoepidermoid carcinoma accounts for less than 5% of head and neck cancers, and 10% of all salivary gland tumors, its localization at the retro molar trigone represents 6.4% of accessory salivary gland tumors [1].our paper reported a case of low grade MEC that sits at the level of the left retromolar trigone, in an 83 year old female.

This case has been reported in line with the SCARE criteria [16].

### 1.1 Case report

**Fig. 1:**
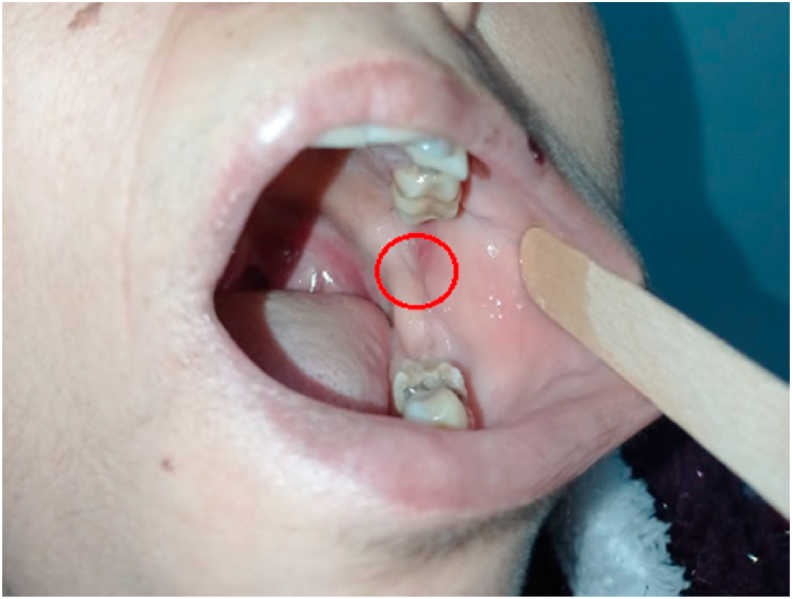
Image showing an erythematous aspect of the left retro molar trigone.

An 83-year-old female, with no previous medical history, reported to our department of oral and maxillofacial surgery for a swelling that had been ongoing for 4 years (Fig. 1), located on the left retromolar trigone, oval, well defined, hard, painless, 15 mm in length, sticking to the mandibular ramus, with an erythematous mucosa opposite.

The patient underwent a biopsy of the lesion, and the anatomopathological assessment revealed a fibrous connective tissue and striated muscle, infiltrated by a tumor proliferation made of cells with rounded or oval nuclei, moderately atypical within an abundant, clear, eosinophilic or sometimes mucoid cytoplasm, the mitosis figures are few, this architecture corresponds to a mucosal squamous cell carcinoma of low grade.

**Fig. 2:**
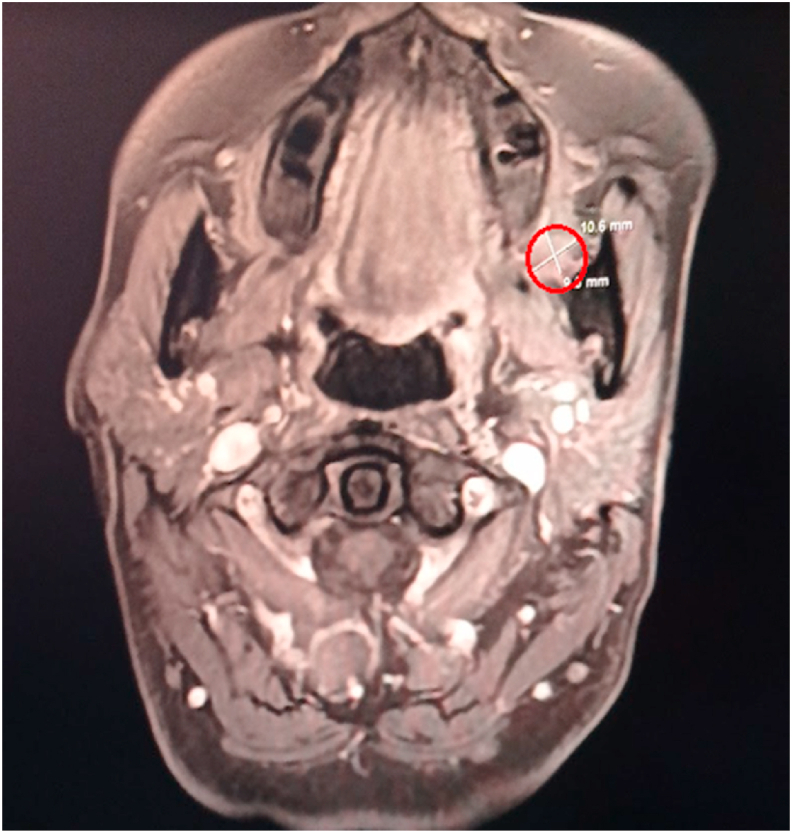
MRI axial section revealing a soft tissue tumor in the left retromolar region.

Cervicofacial MRI showed a poorly limited enhancement area in front of the left inter-maxillary commissure with a 10 mm main axis (Fig. 2), without adenopathy or bone abnormality on the CT scan. The thoracic-abdominal-pelvic CT scan was without any particularities and the lesion was classified as T1N0M0.

Surgery was performed under general anesthesia, and the tumor was removed with a surrounding margin of 1.5 cm. Surgical intervention was performed by our chief of maxillofacial department who has 15 years of operative experience. The postoperative period was simple, and the excisional specimen was sent for anatomopathological analysis, the result of which was in favor of a complete excision of the tumor.

## 2 Discussion

The tumors of the retromolar trigone region represent 12% of neoplasms of the oral cavity [3].They are represented by squamous cell carcinoma in the majority of cases (>95%), adenocarcinoma, adenoid cystic carcinoma, and mucoepidermoid carcinoma which is rare [1].

Mucoepidermoid carcinoma accounts for less than 5% of head and neck cancers, and that 10% of all salivary gland tumors, usually affects the parotid gland 80%, 8–13% of cases the submaxillary gland, 2–4% sublingual gland, The accessory salivary glands remain a rare localization, concerning the localization it can sit at the palate, the oral floor, the oral mucosa, lips and tongue, its localization at the retromolar trigone represents only 6.4% [1].

Mucoepidermoid carcinomas were considered as benign lesions and were called “mucoepidermoid tumor”. The first report on “mucoepidermoid tumors” was by Stewart et al. [2], who divided these tumors into two groups benign and malignant, 8 years later, Foote and Frazell [3] noticed that some patients with “relatively benign” tumors Since then, many authors have adopted the classification of mucoepidermoid carcinoma into three grades, a low grade, an intermediate grade and a high grade. Nowadays, it is possible to study other biological parameters, which recognize the proliferative nature of the tumor, as DNA flow cytometric analysis (DNA index, proliferative fraction), proliferating cell nuclear antigen (PCNA) and Ki-67 proliferation antigen [4].

Histologically, mucoepidermoid carcinomas contain in variable proportion, three cellular elements: squamous cells, mucus-secreting cells and intermediate cells. The composition varies according to the grade of the tumor, according to the WHO classification (2005), these tumors are classified into three grades: low, intermediate and high grade [5]. Although high-grade lesions are malignant and have a poor prognosis [6,7].Other various grading criteria have been suggested, which include the degree of tumor invasion, anaplasia, mode of invasion, degree of maturation of the various cellular components, mitotic index, presence or absence of necrosis, perineural sheathing or vascular emboli [8], the reported case was classified as low grade.

Mucosal squamous cell carcinoma affects mostly women, between the sixth and seventh decade of age [[9], [10], [11]], Low grade tumors develop as a painless, slow-growing swelling, over several years, clinically mimicking a pleomorphic adenoma or other benign tumor, and rarely exceed 5 cm in size, whereas high-grade malignant tumors grow rapidly, are painful, associated with sensitivity or facial motor disorders, infiltrate adjacent tissues, and are associated with adenopathy and distant metastases [11], especially in the lung and bone. In the reported case, the evolution was slow over 4 years, without signs of malignancy.

Regarding imaging, standard radiography, sialography and PET scan yield little information for diagnosis and are rarely indicated for the evaluation of salivary gland masses, but ultrasound can detect lesions smaller than 2 cm in diameter, showing a homogeneous structure with regular boundaries, which may be mistakenly considered benign. CT and MRI give a better visualization of salivary gland masses. CT scans are particularly useful in assessing the location and density of the lesion; they allow differentiation of cystic lesions from solid lesions as well as assessment of boundaries with adjacent structures, including bone and soft tissue [12].

Therapeutically, salivary gland tumors respond well to surgical treatment, the extent of excision depends on the location, size and histo-pathological type. According to the SFORL recommendations, in the absence of adenopathy, curage is indicated for T2-T4 high grade and T4b tumors, it is optional for low grade and T1 high grade tumors. Radiation therapy complements surgery in high-grade MEC stages II, III and IV and in low-grade tumors stages III and IV [13]. Chemotherapy is not recommended for this type of cancer. Radiation therapy is recommended for high-grade CME [6,14]. Chemotherapy is not recommended for this type of cancer tumor [13]. According to Ord and Salama, soft tissue resection with a mucosal margin of 1 cm and preservation of the underlying bone tissue is possible only in cases of low-grade MEC at stage T1 measuring less than 2 cm in diameter and showing no clinical or radiological evidence of bone invasion [15]. According to the authors, these tumors are slow-growing and usually do not infiltrate widely. In the absence of intraoperative findings of bone invasion, the bone is thus left intact [13].

MECs are most properly treated with surgery, the extent of which depends on the location, size, and histopathological grading. Local resection is the treatment option for less aggressive low-grade tumors, while high-grade tumors require wide resection with the involvement of adjacent structures. Treatment of MECs in minor salivary glands is mainly surgical [17]. In our case, the mucosal squamous cell carcinoma was low grade with a TNM classification of T1N0M0 and complete surgical excision with a 1.5 cm margin all around was the treatment of choice.

The prognosis of these tumors depends on the grade and healthy surgical margins, a 5-year survival rate with 92–100% in low grade, 62–92% in intermediate grade and 0–43% in high grade. Clinico-pathological studies have supported that the predictors of morbidity and mortality of salivary gland MEC are: tumor size, histological grade, clinical stage of the disease, presence of perineural engorgement or vascular emboli, lymph node expectation and distant metastasis.

## 3 Conclusion

The mucoepidermoid carcinoma of the accessory salivary glands of the retromolar trigone presents a rare entity, with a clinical picture, prognosis, and therapeutic management, depending on the histological grade. The main therapy is surgical removal combined with radiotherapy essentially for the forms of high grade malignancy.

## Author contribution

Ulrich Opoko: Corresponding author writing the paper. Rachid Aloua: writing the paper. Ouassime kerdoud: writing the paper. Zahra Hmoura: writing the paper. Faiçal Slimani: Correction of the paper.

## Provenance and peer review

Not commissioned, externally peer reviewed.

## Conflicts of interest

Authors of this article have no conflict or competing interests. All of the authors approved the final version of the manuscript.

## Sources of funding

The authors declared that this study has received no financial support.

## Ethical approval

Written informed consent was obtained from the patient for publication of this case report and accompanying images. A copy of the written consent is available for review by the Editor-in-Chief of this journal on request.

## Consent

Written informed consent was obtained from the patient for publication of this case report and accompanying images. A copy of the written consent is available for review by the Editor-in-Chief of this journal on request.

## Registration of research studies

1. Name of the registry:
2. Unique Identifying number or registration ID:
3. Hyperlink to your specific registration (must be publicly accessible and will be checked):

## Guarantor

Ulrich opoko.

Zahra hmoura.
